# Reducing Magnesium within Seawater Used in Mineral Processing to Improve Water Recovery and Rheological Properties When Dewatering Clay-Based Tailings

**DOI:** 10.3390/polym14020339

**Published:** 2022-01-16

**Authors:** Matías Jeldres, Norman Toro, Sandra Gallegos, Pedro Robles, Iván Salazar, Phillip D. Fawell, Ricardo I. Jeldres

**Affiliations:** 1 Departamento de Ingeniería Química y Procesos de Minerales, Facultad de Ingeniería, Universidad de Antofagasta, Av. Angamos 601, Antofagasta 1240000, Chile; hugo.jeldres.valenzuela@ua.cl; 2 Faculty of Engineering and Architecture, Universidad Arturo Prat, Almirante Juan José Latorre 2901, Antofagasta 1244260, Chile; notoro@unap.cl (N.T.); chichined@gmail.com (S.G.); 3 Escuela de Ingeniería Química, Pontificia Universidad Católica de Valparaíso, Valparaíso 2340000, Chile; pedro.robles@pucv.cl; 4 Departamento de Ingeniería Civil, Universidad Católica del Norte, Antofagasta 1270709, Chile; isalazar@ucn.cl; 5 CSIRO Mineral Resources, Waterford, WA 6152, Australia; phillip.fawell@csiro.au

**Keywords:** rheology, clay-based tailings, treated seawater, flocculation

## Abstract

In areas where access to water for mineral processing is limited, the direct use of seawater in processing has been considered as an alternative to the expense of its desalination. However, efficient flotation of copper sulfides from non-valuable phases is best achieved at a pH > 10.5, and raising the pH of seawater leads to magnesium precipitates that adversely affect subsequent tailings dewatering. Seawater pre-treatment with lime can precipitate the majority of magnesium present, with these solids then being removed by filtration. To understand how such treatment may aid tailings dewatering, treated seawater (TSw) was mixed with raw seawater (Rsw) at different ratios, analyzing the impact on the flocculated settling rate, aggregate size as measured by focused beam reflectance measurement (FBRM), and vane yield stress for two synthetic clay-based tailings. A higher proportion of Tsw (10 mg/L Mg^2+^) led to larger aggregates and higher settling rates at a fixed dosage, with FBRM suggesting that higher calcium concentrations in Tsw may also favor fines coagulation. The yield stress of concentrated suspensions formed after flocculation decreased with higher proportions of Tsw, a consequence of lower flocculant demand and the reduced presence of precipitates; while the latter is a minor phase by mass, their high impact on rheology reflects a small particle size. Reducing magnesium concentrations in seawater in advance of use in processing offers advantages in the water return from thickening and subsequent underflow transport. However, this may not require complete removal, with blending Tsw and Rsw an option to obtain acceptable industrial performance.

## 1. Introduction

Mining is a small consumer of water globally, relative to agricultural or forestry industries, but any such consumption can still generate significant social and environmental impacts, particularly for arid areas [1]. Therefore, the industry has made great efforts to optimize water use and recycle it efficiently within their processes. Despite these efforts, the water lost in tailings can still exceed 0.75 tonnes for each tonne of ore treated.

Seawater desalination, usually achieved through reverse osmosis (RO), can ensure an adequate supply for processing operations. However, the installation of desalination plants is the subject of economic and environmental debates. For example, it requires the release of concentrated brine to the environment, and disposal methods include surface water discharge, sewer discharge, or deep well injection. Differences in salinity, pH, temperature, and dissolved species can negatively impact marine flora and fauna [2,3]. RO processes require electricity, which is generally supplied from fossil fuels; hence, the carbon footprint involved in seawater desalination plants is relative, varying from 0.4 to 6.7 (kg CO_2_eq/m^3^) [4].

Alternatively, the direct use of seawater in processing avoids the need to construct a desalination plant. However, it would still require new technologies, as older plant infrastructure will suffer damage from high salinities and, in particular, high chloride levels [5]. Operationally, the consequences in the concentration stages are potentially significant. The flotation of copper minerals is typically carried out at a pH above 10.5, to depress non-valuable sulfides such as pyrite, which will otherwise float and contaminate the concentrate [6,7]. However, solid Ca/Mg complexes can arise when using seawater at a high pH, causing a considerable increase in lime consumption and low molybdenite recovery [8,9]. The process can then only be applied at a natural pH, which requires additional reagents (e.g., sodium metabisulfite, cyanide, sulfur dioxide) to improve the quality of the concentrates, but these may also be harmful to the environment and human health (in fact, the use of cyanide-based depressants has been banned in several countries [6]).

The consequences of seawater use during processing on subsequent thickening operations can also be significant, with recent studies showing that the formation of precipitates at high pH leads to reduced settling rates for slurries flocculated by polymeric bridging. For example, Ramos et al. [10] studied the impact of seawater on the flocculation of synthetic mine tailings across a range of pH conditions, finding that magnesium precipitates formed at pH > 10.3 have a high affinity with the flocculant, and their presence competes with the mineral surface for polymer adsorption, greatly reducing the extent of aggregation. Excessively high dosages are then required to effectively aggregate the high number of precipitate and tailings fine particles to achieve an industrially acceptable settling performance. Calcium did not adversely affect the flocculation process within the pH range considered (pH < 11.1).

The industry is increasingly being forced to consider new methods or technologies that adapt to the challenges of a highly-saline environment. In response to this problem, Castro [11] proposed removing divalent cations of seawater before it is used in froth flotation, achieving a promising performance in molybdenite recovery. Then, Jeldres et al. [12] examined the use of seawater treated with lime, to reduce its magnesium concentration. The authors increased the settling rates and reduced supernatant turbidity at pH 11, obtaining values that even exceeded the performance at the natural pH. Studies that complement this strategy have considered divalent ion removal through an atmosphere rich in carbon dioxide [13] or a biotechnological approach [14].

Settling rate and supernatant turbidity are key flocculation performance parameters in test work for solid–liquid separation, as they can indicate the solid residence time inside the thickener and the quality of the recirculated water, respectively. However, caution should be exercised if only using these two parameters. The thickened tailings rheological behavior is also helpful for evaluating potential thickening performance [15,16,17,18]. Yield stress corresponds to the minimum effort needed to make a material or high-solid suspension flow, and it is the most common rheological parameter used to characterize mine tailings after thickening. This is linked to the energy required to pump suspensions from the thickener discharge to the storage facilities. Subsequent deposition strategies are also strongly related to the yield stress [19,20].

Rheological behavior is well known to be influenced by colloidal particle aggregation. Johnson et al. [21] interpreted the yield stress of alumina, zirconia, and kaolin suspensions through different types of interaction forces. Similar trends can be seen with the aggregation induced by polymers, with Zhou et al. [22] finding the yield stress of silica flocculated with polyelectrolytes has a direct relationship with adhesion forces, and strongly influenced by the pH, salinity, and polymer characteristics. Jeldres et al. [23] studied the influence of salinity on the viscoelastic properties of kaolin sediments, settled after flocculating with a high molecular weight anionic polyacrylamide. They found that low levels of added salt elevated the yield stress, but after further increases in salinity, they saw a distinct maximum in the rheological parameters. This was interpreted with the DLVO theory, highlighting that a high ionic concentration compresses the double layer of particle surfaces and eliminates repulsions between the charged functional groups on the polymer. Neelakantan et al. [24] analyzed the effect of hydrodynamic conditions during kaolinite flocculation, observing that an increase in mixing intensity produced a considerable reduction in the size of aggregates and the sediment yield stress, thought to be the elimination of retained water within porous aggregates because of shear-induced fragmentation. This would create a dramatic reduction in the apparent volume of solids in the suspension.

A direct relationship between the settling rates of tailings in seawater at a natural pH with the yield stress of the resulting sediment was found by Jeldres et al. [25]. Aggregate size and structure played a significant role in determining the rheological behavior, with an exponential relationship between yield stress and the aggregate fractal dimension. There have been a few studies of tailings rheology at an alkaline pH, at which some divalent cations can be precipitated as hydroxides. Avadiar et al. [26] stated that calcium complexes have a high affinity for kaolinite particle surfaces and promote aggregation, implying an increased yield stress. Analyzing the impact of the hydrolysis of calcium and magnesium cations on quartz, alumina, and kaolin tailings, they found the complexes could adhere to all surfaces, causing a significant elevation in zeta potential [27]. Jeldres et al. [28] showed that kaolin could adsorb a substantial proportion of the magnesium within seawater, moderating precipitate formation at high pH; this phenomenon would be significant at high clay concentrations.

The studies described above provide valuable insights into clay surface behavior in saline media under various conditions. However, they do not directly consider the rheological consequences of solid precipitate formation and their interactions with polyelectrolytes, which are expected to be heightened in clay-based tailings, due to the greater porosity of their aggregate structures. In particular, using seawater treated to reduce its magnesium concentration in the processing of copper ores at a more desirable high pH has potential implications for achieving better dewatering and rheological outcomes from the resulting tailings. In this study, treated seawater (Tsw) was mixed with raw seawater (Rsw) in different proportions, analyzing the impact on the settling rate and yield stress for a synthetic tailings suspension. The analysis was complemented by characterizing both particles and aggregates in real-time during the flocculation process by focused beam reflectance measurement (FBRM).

The results of this work are of particular interest for mining industries located in arid zones, with little access to water resources but with the potential availability of seawater. The study route shows that seawater with magnesium removal is a promising alternative to meet operational challenges in mineral concentration circuits.

## 2. Methodology

### 2.1. Materials

Seawater was collected from the San Jorge Bay in Antofagasta (Chile) and filtered at 1 μm using a UV filter system to eliminate all bacterial activity. Its chemical conductivity was 50.4 mS/cm. The concentrations of the main cations within the seawater were determined by atomic absorption spectrometry to be 10.82 g/L Na^+^, 1.42 g/L Mg^2+^, 0.40 g/L Ca^2+^, and 0.40 g/L K^+^; Cl^−^ (19.61 g/L) was determined by argentometric titration, while HCO_3_^−^ (0.15 g/L) was determined by acid-base titration.

**Figure 1:**
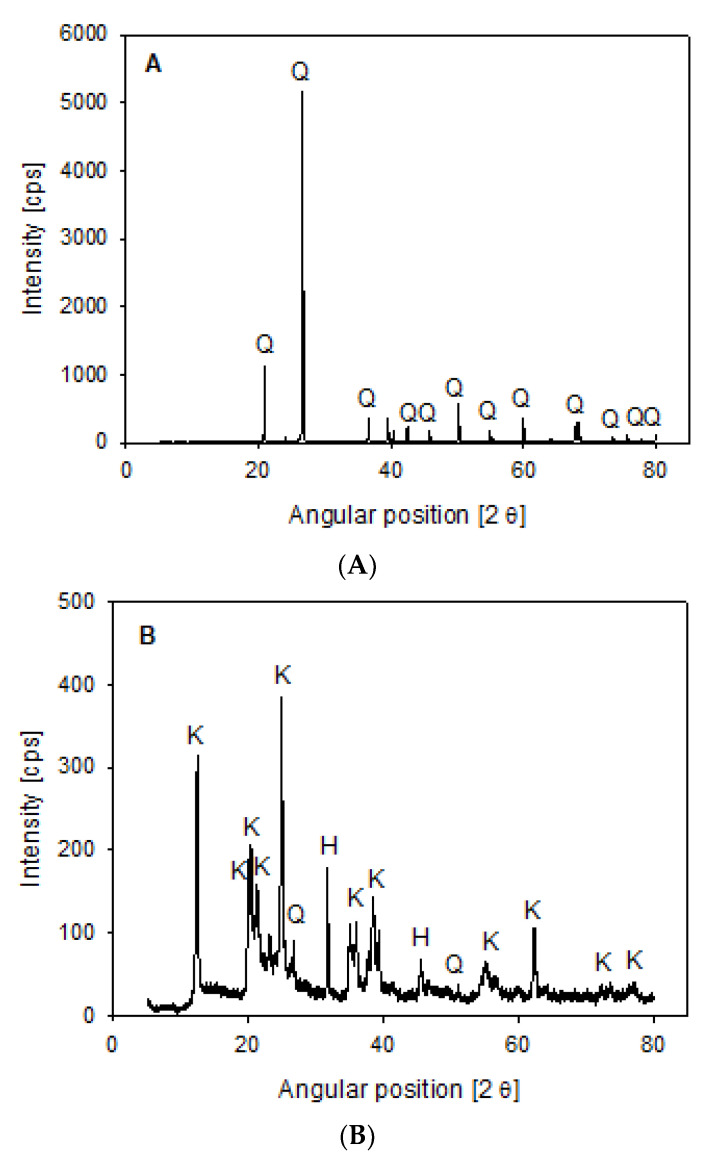
X-ray diffraction of quartz (**A**) and kaolin (**B**) powder, showing kaolinite (K), halite (H), and quartz (Q).

The synthetic tailings were composed of quartz and kaolin purchased from Donde Capo (Santiago, Chile) and Ward’s Science (Rochester, NY, USA). XRD analysis was performed on a Bruker D8 ADVANCE X-ray diffractometer (Bruker, Karlsruhe, Germany) using the Powder Diffraction File of 2020 ICDD (International Center for Diffraction Data) software TOPAS (version 5, Bruker, Billerica, MA, USA). Quartz presented 99% SiO_2_ (Figure 1A), while kaolin was mainly made up of kaolinite (<80%) and halite (>10%), with SiO_2_ (1–10%) as a minor phase (Figure 1B).

**Figure 2:**
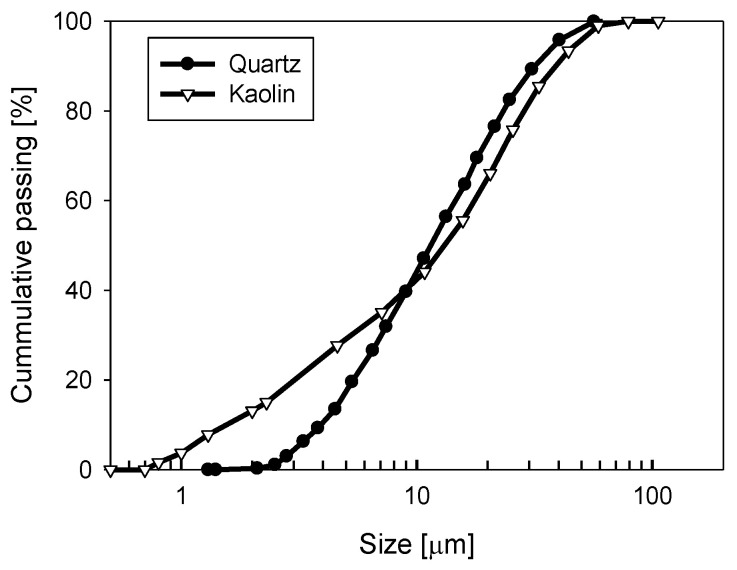
Particle size distribution for quartz and kaolin in distilled water at natural pH. The particles were dispersed by mechanical stirring overnight.

Volume-weighted particle size distributions (PSDs) were obtained using a Microtrac S3500 laser diffraction analyzer (Verder Scientific, Newtown, PA, USA). As shown in Figure 2, the PSDs for both phases were quite similar, with the main difference observed in the d10 values (sizes representing the smallest 10% of the particles by volume), being 1.8 and 3.8 µm for kaolin and quartz, respectively.

The flocculant used throughout was the commercial anionic acrylamide/acrylate copolymer powder product SNF704 (supplied by SNF, Santiago, Chile), which has an intrinsic viscosity of 120 L/g in distilled water and 19 L/g in seawater. The lower intrinsic viscosity value in seawater responds to polymer coiling in a saline medium, limiting its ability to form hydrogen bonds. The soluble polymer has a high molecular weight (>18 × 10^6^ g/mol). This flocculant is typically used in thickening operations in the mining industry [10].

Stock solutions (1 g/L) were prepared in distilled water and kept refrigerated for a maximum period of 2 weeks. Diluted solutions (0.1 g/L) were prepared daily from the stock solution for experimental tests and discarded 24 h after preparation. The reagent used to form the magnesium precipitates was lime (provided by CBB Cales, Antofagasta, Chile). The reagents had a purity higher than 98%.

### 2.2. Magnesium Removal Treatment

To effectively remove magnesium cations from seawater, lime powder was added to obtain a concentration of 0.06 M and mixed with mechanical stirring for 20 min. This generated a highly alkaline environment (pH 11), leading to chemical reactions that produced solid magnesium/calcium complexes [29,30,31], as summarized in Equations (1)–(4). The solubility constants are shown in Table 1.

(1) $$\mathrm{Ca}{\left(\mathrm{OH}\right)}_{2}+{\mathrm{Mg}}^{2+}⟷\mathrm{Mg}{\left(\mathrm{OH}\right)}_{2}+{\mathrm{Ca}}^{2+}$$

(2) $${\mathrm{Ca}}^{2+}+2{\mathrm{OH}}^{-}⟷\;\mathrm{Ca}{\left(\mathrm{OH}\right)}_{2}$$

(3) $$\mathrm{Ca}{\left(\mathrm{OH}\right)}_{2}+{\;\mathrm{SO}}_{4}^{2-}+2H_{2}O⟷{\mathrm{CaSO}}_{4}\cdot 2H_{2}O+2{\mathrm{OH}}^{-}$$

(4) $$\mathrm{Mg}{\left(\mathrm{OH}\right)}_{2}+{\;\mathrm{SO}}_{4}^{2-}⟷{\;\mathrm{MgSO}}_{4}+2{\mathrm{OH}}^{-}$$

**Figure 3:**
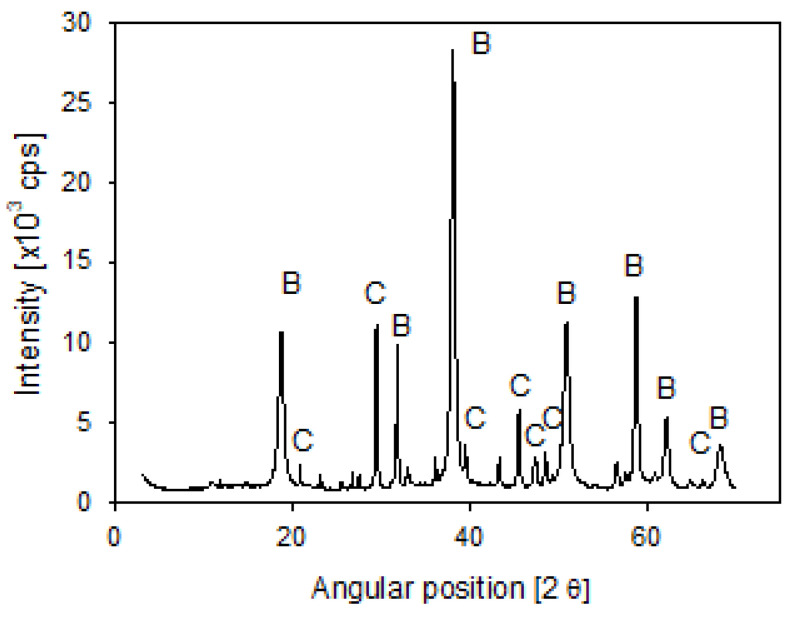
X-ray diffraction of seawater precipitates, showing brucite (B) and calcite (C).

Any such solids were subsequently removed by vacuum filtration. XRD analysis of the collected solids (Figure 3) indicated that the precipitates formed by the addition of lime contained brucite and calcite.

The magnesium concentration in the treated seawater was then measured by inductively coupled plasma mass spectrometry (ICP-MS) and confirmed to be 10 mg/L, representing 97% removal.

### 2.3. Aggregate Characterization

**Figure 4:**
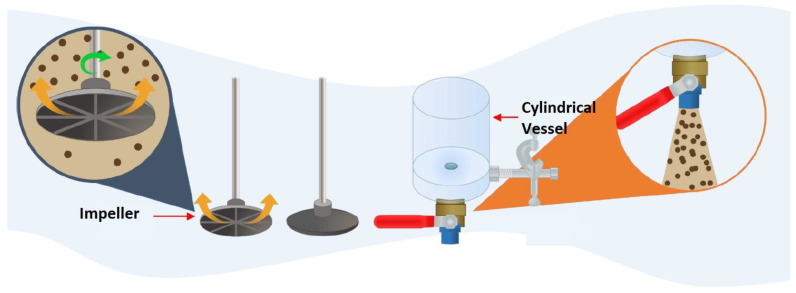
Schematic representation of experimental setup for settling tests.

Flocculation of the synthetic tailings suspensions at the selected quartz/kaolin ratio was achieved in a 1 L capacity cylindrical vessel, 100 mm in diameter, with an 80 mm diameter PTFE turbine impeller at the end of a vertical axis (4 mm diameter) used to keep particles suspended. The impeller base was positioned 20 mm above the bottom surface of the container (Figure 4). The required solid mineral phases were added to a fixed volume of seawater at the desired Rsw/Tsw ratio, to achieve a mass of 270 g. The subsequent volume of the flocculant solution to give the required dosage (expressed as grams of flocculant per tonne of solids, e.g., g/t) and a volume of water was together then adequate to ensure a total suspension mass of 300 g. This methodology maintained the same solid concentration (10 wt%) for all flocculation experiments.

The combined Rsw-Tsw liquor was mixed with the synthetic tailings at 600 rpm for 30 min, with the pH adjusted to 11 by lime addition. Subsequently, the agitation was reduced to 220 rpm, and the flocculant was added at the required dosage. The evolution of aggregate size was measured with the FBRM system (Particle Track E25, Mettler Toledo), which consists of a processing unit and a probe (19 mm diameter) with a sapphire window (14 mm diameter) at the measurement tip. The probe was inserted vertically into the reaction vessel, 10 mm above the stirrer and 20 mm off-axis.

The FBRM probe features a laser focused through the sapphire window and scans a circular path at a tangential velocity of 2 m/s. The beam allows suspended solids in the focal plane to generate backscattered light. A chord length is obtained from the duration of any high backscattered light intensity and the laser’s speed. The FBRM software processes recorded data into histograms of counts corresponding to chord lengths in selected channel sizes, ranging from 1 μm to 1 mm as quickly as every 2 s. In this case, the chord length distributions (CLDs) represent 100 channels in the full range, but the histograms are presented as line graphs for easy comparison. Two distributions were analyzed: the unweighted CLD that offers greater sensitivity to changes in the fines region and the length square weighted CLD mainly influenced by the contribution of coarse particles and aggregates. The measured raw data was post-processed in ‘primary’ mode to better detect dispersed fine particles.

### 2.4. Settling Tests

To determine initial settling rates, the flocculation procedure described above was followed up, to the point of dosing with flocculant. After 20 s of flocculation under the applied mixing, the suspension was transferred through the bottom of the 1 L vessel into a settling cylinder (35 mm internal diameter) that could be stoppered (Figure 4). The entire contents were slowly inverted three times, and the cylinder was immediately placed on a flat surface, with the fall of the liquid–solid interface, then monitored visually for 5 min, recording its height at known intervals. The experiments were repeated twice and the results presented in this work are the average values.

### 2.5. Yield Stress

Rheological behavior was characterized on an Anton Paar MCR 102 rheometer (ANAMIN Group, Santiago, Chile), and the data was processed using RheocompassTM Light version software (ANAMIN Group, Santiago, Chile). A vane-in-cup configuration (model ST22-4V-40, Anton Paar, Graz, Austria) was used, with 2.2 and 4.2 cm diameters, respectively.

For rheological characterization, suspensions for two distinct synthetic solids tailings compositions were prepared in different water types (mixtures of Rsw with Tsw). Tailings 1 and Tailings 2 were intended to represent low and high clay contents, respectively. The rheological study sought to represent thickener underflow characteristics; therefore, the suspensions were prepared with solid concentrations that reached up to 70 wt%. A single flocculant dosage was considered for each system, chosen according to the settling experiments, with the dosage providing a settling rate of 20 m/h used. Consequently, the dosage varied according to the type of water and tailings (see details in Table 2).

Prior to flocculant addition, a 70 wt% stock suspension was prepared for the selected combination of synthetic tailings solids and type of water (8 systems in total). This was dispersed with mechanical stirring at 500 rpm for 2 h; then, a 300 mL portion was taken and diluted with the respective water to achieve the solid target concentration. Stirring this suspension at 200 rpm, the flocculant solution was added, and stirring was maintained for 5 min. Subsequently, 120 mL of the suspension was extracted with a syringe and added to the rheometer cup.

The yield stress was detected in the logarithmic representation of the deformation (γ) over the shear stress (τ). Up to specific shear stress, the relationship between γ and τ is constant, representing the range of elastic deformation. At the end of this range, irreversible deformation occurs with increasing shear stress, resulting in sample flow and a steeper curve slope. To determine the elastic limit in the logγ/logτ diagram, the change of slope in the measurement curve was analyzed with the help of two tangents. The stress where the tangents’ intersection occurs is considered the yield stress. The measurement was carried out in a logarithmic time ramp with an initial duration of 60 s and a final duration of 1 s, linearly increasing the shear stress value with intervals of 1 Pa.

## 3. Results and Analysis

### 3.1. Sedimentation

**Figure 5:**
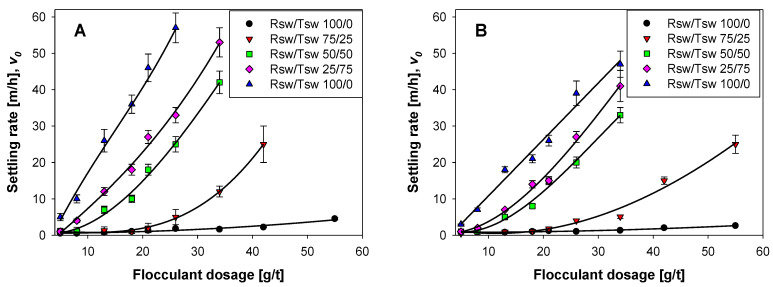
Effect of flocculant dosage on the settling rate of synthetic tailings, using raw seawater (Rsw) and treated seawater (Tsw) at different proportions. (**A**): Tailings 1 with quartz/kaolin at 90/10 ratio; (**B**): Tailings 2 with quartz/kaolin at a 75/25 ratio. Solid concentration: 10 wt%, pH 11.

Figure 5 shows the impact of the flocculant dosage on the settling rate of the clay-based synthetic tailings prepared in mixtures of Rsw and Tsw at different proportions. When using 100% Rsw, the flocculant dosage did not significantly impact the settling rate. Only a slight increase was observed at dosages greater than 40 g/t; however, it did not exceed 5 m/h within the range of dosages studied (5–55 g/t). The magnesium concentration prior to pH adjustment, in this case, was at its highest (~1.4 g/L), and the significant inhibition of flocculation was consistent with the results of Ramos et al. [10]. They attributed the low settling rate in seawater at alkaline pH to magnesium precipitates.

A higher proportion of Tsw leads to a lower amount of magnesium in the suspension before pH adjustment to pH 11, consistently enhancing flocculation. For example, when 100% Tsw (10 mg/L of Mg) was used, a settling rate over 40 m/h was obtained for Tailings 1 (Qz/Kao 90/10), flocculated at a dosage of 21 g/t. In this system, the flocculant interacts predominantly with the surfaces of the two tailings mineral phases. When water with equal proportions of Tsw and Rsw is used, the magnesium in solution before adjusting the pH to 11 was ~700 mg/L. Consequently, the dosage required to achieve a settling rate of 20 m/h with Tailings 1 at the high pH effectively doubled, reflecting the additional solids present within the suspension.

The increased concentration of clay within Tailings 2 adversely affected the flocculation performance. As observed in Figure 5, increasing the proportion of kaolin within the synthetic tailings from 10 to 25% reduced the settling rates achieved at any applied dosage for all the systems studied. In part, this reflects the finer size of kaolin relative to quartz (Figure 2), with a much higher number of particles and a large specific area for the clay, increasing flocculant demand. In addition, the plate-like shape of the clay particles and the preference for flocculant to adsorb on the minor edge faces [32] leads to very low flocculated aggregate density, which can further hinder settling at moderate solid concentrations.

Although Figure 5 clearly shows that the flocculation performance improved with the degree of magnesium removal achieved prior to pH adjustment, it is noted that complete removal may not be necessary to still attain acceptable performances for most industrial purposes. Required settling rates will depend upon the unit’s thickener diameter and volumetric throughput, but the majority of copper tailings thickeners would comfortably operate with settling rates >10 m/h, with values >20 m/h more than adequate in all applications. At high removal (Mg = 10 mg/L), the dosages required were very low, and large, fast settling aggregates were readily achieved, but this was still possible with up to 50% Rsw, without a massive penalty in terms of the applied dosages.

### 3.2. Particle and Aggregate Characterization

While FBRM has proved to be an important tool for the real-time monitoring of aggregation states during flocculation at industrially relevant solid concentrations, it can also provide useful insights into unflocculated feed suspensions. This is because the unweighted chord length distributions (CLDs) offer a much higher sensitivity to particle number than traditional volume-based particle size distributions (as shown in Figure 2), and particle number can be a more useful indicator of likely flocculation responses [33].

**Figure 6:**
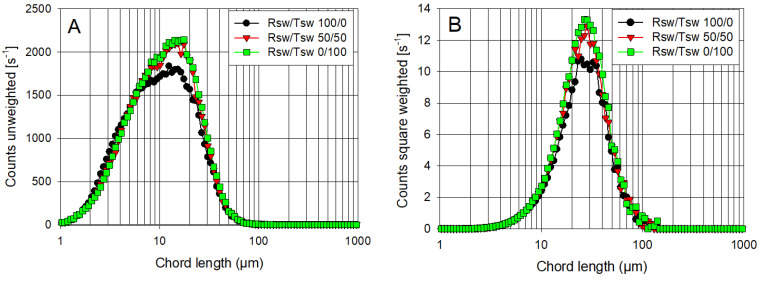

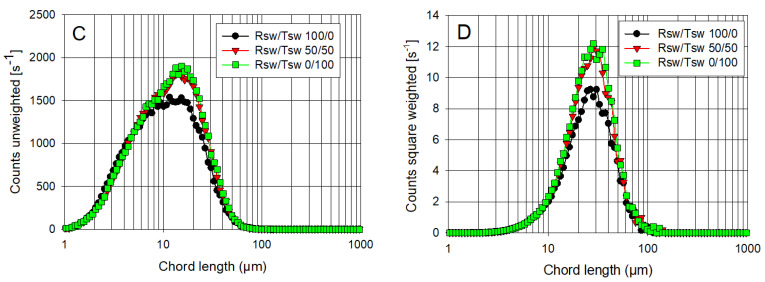
Unweighted and square-weighted chord length distributions (CLD) for unflocculated slurries. (**A**,**B**): Qz/Kao 90/10; (**C**,**D**): Qz/Kao 75/25.

Figure 6A,B shows the unweighted and square-weighted CLDs, respectively, for unflocculated Tailings 1 (Qz/Kao 90/10), when suspended in Rsw, Tsw, and the 50/50 mix of both (Msw) after adjustment to pH 11. The suspension in Rsw shows a peak near 15 µm in the unweighted CLD (Figure 6A), but there is a broad shoulder to the left, with the majority of the counts in the distribution being <10 µm. This region is expected to be dominated by the contribution from the clay phase, and the low counts observed <2 µm need to be viewed with caution, given that FBRM has reduced sensitivity at the shortest chord lengths; there will undoubtedly be clay particles within the suspension <1 µm in size, although they are likely to be aggregated to some degree at high ionic strength.

With the use of 50% or 100% treated seawater (Msw and Tsw, respectively), this peak increased in height, while there was a corresponding smaller, but still distinct, reduction in counts <6 µm. Several factors are likely to be at play in these responses. The CLD with Rsw will include the contribution of phases precipitated when the pH is raised to 11, which will be reduced for Msw and effectively eliminated for Tsw. This reduction in overall particle number in suspension (which may still manifest in the observed counts <6 µm) is masked by increased coagulation, to give small aggregates, but serves to substantially reduce the available surface area then presented to the dosed flocculant, consistent with the enhanced settling rates observed in Figure 5.

The primary particle aggregation responses can be explained by changes in the solution cations concentration with the type of water used, as summarized in Table 3. The pH of the suspension in Rsw was adjusted to 11 with NaOH. Virtually all magnesium then precipitates as brucite, while a proportion of the calcium present in seawater precipitates as calcite [34]. This led to the low residual solution concentrations of both divalent cations (70 and 335 mg/L for Ca^2+^ and Mg^2+^, respectively). In Tsw, magnesium was selectively removed in advance through treatment with lime. Although the solution concentration of magnesium is low (10 mg/L), there is a much higher presence of calcium (2210 mg/L). The higher divalent cation concentration for Tsw (and hence Msw) relative to Rsw would favor greater coagulation of the primary particles.

The corresponding volume-weighted CLDs (Figure 6B) support particle aggregation being enhanced when Tsw is used, with the peak increase in height but not being displaced from ~28 µm. This is consistent with the coagulated aggregates formed from the fine particles being quite weak, and only able to attain sizes comparable to that of the largest primary particles within the synthetic tailings solids under the applied shear conditions.

Tailings 2 consisted of a greater proportion of clay (Qz/Kao 75/25), and the unweighted CLD in Rsw (Figure 6C) displayed a higher peak intensity than Tailings 1. This is expected, given that the fine clay mineral generates more particles than quartz when considering the same mineral mass, as shown in Figure 1. The observed trends on changing the water composition were also the same, with the reduction in counts <6 µm even more apparent. Similarly, the volume-weighted CLDs in Figure 6D only differ from Figure 6B in their peak height.

**Figure 7:**
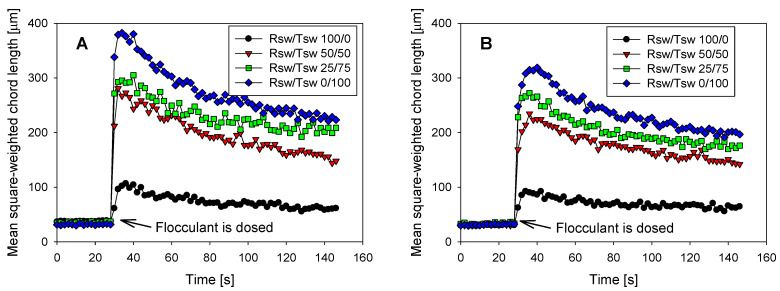
Flocculation kinetics (dosage 20 g/t) as monitored by FBRM for synthetic tailings made up of different ratios of raw seawater (Rsw) and treated seawater (Tsw). (**A**): Qz/Kao 90/10; (**B**): Qz/Kao 75/25.

Subsequent flocculation leads to much larger shifts in the chord length response, with the impact of water composition far more obvious. Figure 7 represents the kinetics of the flocculation process for both synthetic tailings at a fixed flocculant dosage, plotting the mean aggregate size as captured by FBRM as a function of reaction time. A rapid rise in size was observed on dosing the flocculant solution (~30 s), with a peak size attained within just a few seconds in all cases. This peak increased as the proportion of magnesium within the water and clay within the synthetic tailings was reduced. Short reaction times will favor large aggregate formation, but these are intrinsically fragile structures, and therefore sizes will decline with time as the aggregates either break or reconform as they collide under the applied shear [35]. The aggregate sizes appear to approach a plateau value at longer times, although, in reality, breakage is irreversible in the absence of fresh flocculant being added, with a very gradual decline still expected.

Good correlations were observed between mean aggregate sizes as monitored in situ by FBRM and subsequently measured settling rates [36], but FBRM was more sensitive than bulk settling rates to the onset of aggregation [33]. The latter was confirmed for the current system, with Figure 5 indicating that settling rates for both synthetic tailings were negligible in Rsw at all but the highest flocculant dosages applied, whereas Figure 7 shows that aggregation at 20 g/t was evident, if substantially suppressed, relative to the use of water with reduced magnesium contents.

**Figure 8:**
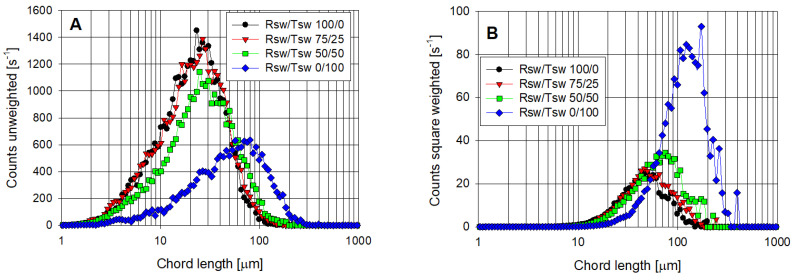
(**A**) unweighted and (**B**) square-weighted chord length distributions from the flocculation of synthetic tailings in raw seawater (Rsw) and treated seawater (Tsw). Tailings 2 (Qz/Kao 75/25), solid concentration 10 wt%, flocculant dosage 20 g/t, flocculation time 90 s (after dosing).

Focusing on the synthetic tailings with the highest clay content (Tailings 2, Qz/Kao 75/25), Figure 8 contrasts the unweighted and square-weighted CLDs at 90 s after the flocculant is dosed in water with different ratios of Rsw and Tsw. While the unweighted CLD in 100% Rsw shows a peak intensity only slightly lower than that prior to flocculation (Figure 6C), there was a substantial reduction in the counts <10 µm, indicative of the solids within this finer region of the CLD (almost certainly small, coagulated aggregates themselves) being captured into larger but still only moderately sized aggregates. The corresponding volume-weighted distribution (Figure 8B) shows a peak between 40 and 50 µm, representing a small shift from the unflocculated peak near 30 µm (Figure 6D).

Figure 8 shows only slight shifts to larger chord lengths on introducing 25% Tsw into the water used with the synthetic tailings. This is consistent with the settling rate response to dosage (Figure 5B), with the moderate additional aggregation detected at 20 g/t insufficient to give a discernable improvement over the Rsw results. However, higher dosages led to a steady elevation in settling rates, suggesting a clear benefit from a reduced presence of fine particles (and their high available surface area) from precipitated magnesium.

The unweighted CLD for Tailings 2 in 50% Tsw prior to flocculation displayed a higher peak than for 100% Rsw (Figure 6A), which reflected fewer precipitated fines in suspension and the higher calcium concentration favoring fines coagulation. This further reduction in fines had a more substantial impact on the level of aggregation observed by FBRM on flocculation at a low dosage (Figure 8), and it is important to note that this was sufficient to ensure fast settling rates were achieved at higher dosages, even with the high clay content (Figure 5B). Tailings 2 prepared in 100% Tsw had practically no magnesium ions present, minimizing precipitates that can hinder flocculation performance, which is readily seen in the FBRM response, and this clearly reduced the dosages required to attain high settling rates. Likely, the differential in performance for flocculation of Tailings 2 at 50% and 100% would be much greater at higher solid concentrations.

**Figure 9:**
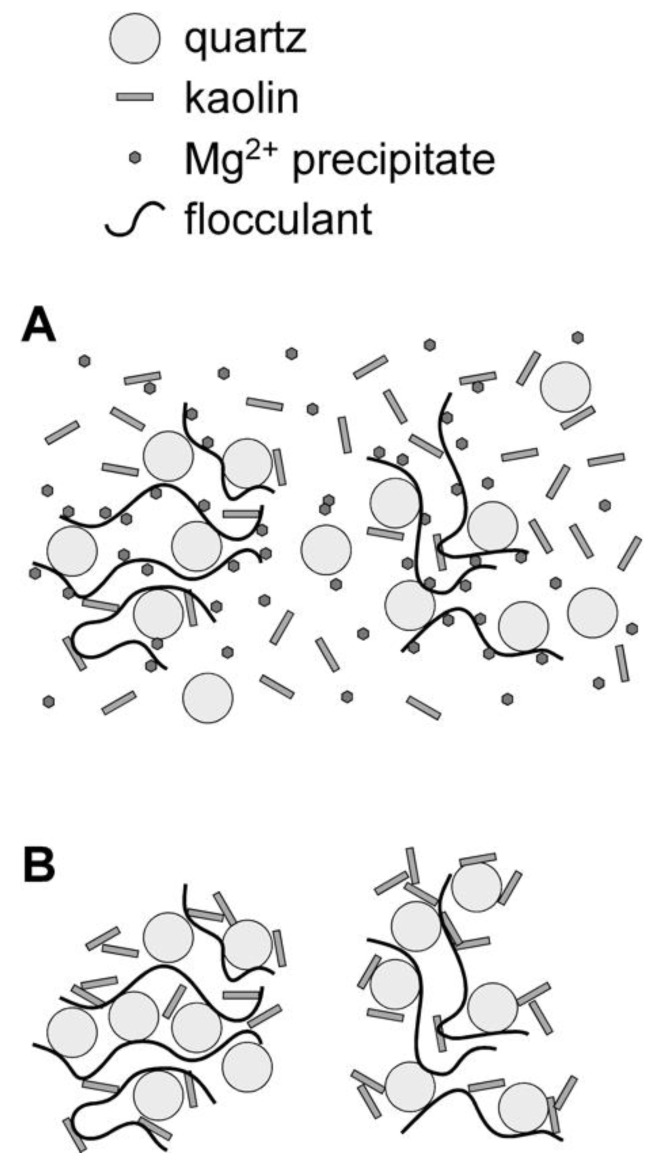
Schematic representation of aggregate formation in synthetic tailings containing quartz and kaolin. (**A**): flocculation in Rsw, (**B**): flocculation in Tsw.

Figure 9 shows how the type of water and the formation of precipitates affects the flocculation efficiency and the resulting aggregate structures. When the system contains magnesium precipitates (main brucite), the flocculant is less effective in capturing the quartz or kaolin phases of synthetic tailings, interacting preferentially with the surfaces of the precipitate at high pH, as demonstrated by the predictions of molecular dynamics of Quezada et al. [34]. The researchers proposed two adsorption mechanisms between the flocculant and the minerals: (i) hydrogen bridges and (ii) cationic bridges.

Hydrogen bonds occur when two electronegative groups interact through a hydrogen atom bound to one of them, generating a stronger interaction than a typical dipole. The results of Quezada et al. showed that the flocculant generates a much higher proportion of bonds with brucite than with the rest of the minerals. The main interactions are through bonds between the hydroxide group on the brucite’s surface and the acrylamide group’s nitrogen.

Cationic bridges arise from two anionic groups interacting through a dissolved counterion that balances charge. These interactions are more stable and predominate over hydrogen bonds, a product of the high concentration of cations in the medium, considering that the liquid is seawater. The amount of ionic bridges is similar between brucite and kaolinite. The cationic bridges of brucite flocculant mainly occur between the hydroxide group on the solid surface and the flocculant’s acrylamide group. These interactions are relatively stable because the cations in seawater are adsorbed between three OH groups and are exposed to interactions with COO- (charged groups of HPAM).

The precipitate phase also introduces a significantly larger surface area into the system, increasing the demand for flocculants. Combining these factors leads to small aggregates with porous structures and poor overall fine capture (Figure 9A). This behavior only intensifies as the clay content increases, going from Tailings 1 to 2, as this further increases the proportion of fines within the solid phases present.

With magnesium precipitates absent when using Tsw instead of Rsw, the flocculant is not then inhibited from interacting freely with the particles that make up the tailings, and there are far fewer fines within the suspension (Figure 9B). Consequently, the same flocculant dosage can form larger aggregates, and the fines capture will be more efficient; fewer fines being incorporated within the aggregates is also likely to result in a higher effective aggregate density.

### 3.3. Rheological Behavior

**Figure 10:**
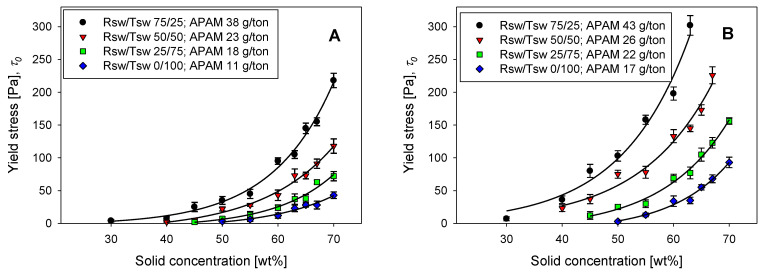
Yield stress of clay-based synthetic tailings flocculated in a range of solid concentrations, using raw seawater (Rsw) and treated seawater (Tsw) at different proportions. (**A**): Tailings 1 with quartz/kaolin at 90/10 ratio; (**B**): Tailings 2 with quartz/kaolin at a 75/25 ratio.

The yield stress of flocculated synthetic tailings in four water compositions is plotted as a function of solid concentration in Figure 10. A different flocculant dosage was applied in each system, such that an initial settling rate of 20 m/h was achieved, considered representative of the demand on flocculation within industrial tailings thickeners. Note that results for suspensions in 100% Rsw are not shown, because they failed to achieve the target settling rate within a practical dosage range.

The expected exponential relationship between yield stress and solid concentration was observed for all tailings and water compositions, with the responses only varying in terms of the onset in the elevation of yield stress and the magnitude attained. A higher proportion of Tsw consistently shifted the yield stress to the right, i.e., to higher solid concentrations. Several factors combined to give this effect:

(i.) The presence of precipitates implies a higher proportion of fine solids in the suspension. The solid concentrations in Figure 10 were calculated based on the synthetic tailings solids only, with the total mass of the formed precipitates themselves only then accounting for a shift in the order of 0.2–0.3 wt%. However, rheological responses within mineral systems are dominated by the fines fraction [16,17], and the capture of such fines during flocculation, as illustrated in Figure 9, will also lead to lower density aggregates, with higher effective aggregate volumes for a given mass.
(ii.) As the magnesium content within the water used to prepare the suspension was lowered, the flocculant dosage then required to achieve the targeted settling rate (20 m/h) reduced, more than halving, going from 25 to 100% Tsw. Therefore, there is a direct effect on the amount of flocculant in each system, with the impact of higher dosages during initial flocculation persisting after sedimentation and consolidation to impact the strength of the particle networks within the high solids suspensions [25,37,38].

**Figure 11:**
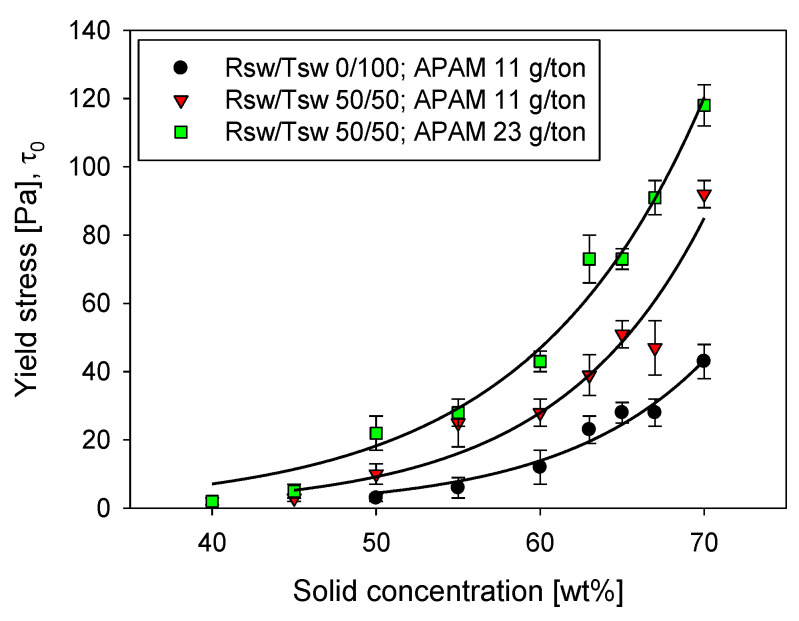
Yield stress of clay-based synthetic tailings (Tailings 1 with quartz/kaolin at 90/10 ratio) flocculated in a range of solid concentrations, using treated seawater (Tsw), and a 50/50 blend of raw seawater (Rsw) and Tsw (Msw).

Both factors are more clearly reflected in Figure 11. The yield stress response at a fixed dosage (11 g/t) for Tailings 1 in 100% Tsw was consistently lower across the solid concentration range when contrasted with suspensions in water with Tsw and Rsw in a 50/50 ratio. The effect of flocculant dosage is also evident for the latter system, with an increase from 11 to 23 g/t significantly elevating the yield stress for solid concentrations above 50 wt%.

Changes in the proportion of clays also altered the rheological properties of the suspensions. As shown in Figure 10, an increase from 10 to 25% kaolin on going from Tailings 1 to 2 meant a considerable increase in yield stress; for example, with 100% Tsw, the yield stress rose from 43 to 93 Pa at 70 wt%, while the use of a 50/50 mixture of Tsw and Rsw the yield stress increased from 91 Pa at 70 wt%, to well in excess of 226 Pa at a lower solid concentration (67 wt%). This reflects not only the clay’s smaller size but also a plate-like shape and heterogeneous surface structure, increasing both particle–particle and particle–fluid interactions. In seawater, the ions adhere to the particles’ surfaces by electrostatic attraction, favoring coagulation prior to polymer-induced flocculation, resulting in more extensive particle networks at high solids.

The above results offer promise in the search for alternative water sources for mineral processing in areas of freshwater scarcity, seawater may be accessible, but its direct use has the potential for detrimental effects on flowsheet performance. While the advantages of using seawater with a reduced magnesium content have previously been identified for froth flotation [8,11] and flocculation in thickening operations [12,13], the present study suggests there may also be substantial improvements in slurry fluidity, offering a more economical strategy for pumping thickened tailings and possibly the opportunity to thicken to high solid concentrations, improving the efficiency of water use and advancing to effective closure of water circuits.

## 4. Conclusions

This research considered the implications following mineral processing at a high pH using seawater treated to specifically reduce its magnesium content on tailings flocculation and the subsequent flow properties of the thickened suspensions. Treated seawater (low-magnesium) was prepared by reaction with lime and the solids were removed prior to use; varying magnesium levels were examined by mixing this treated seawater with raw seawater (high-magnesium) at different proportions.

When flocculation and sedimentation of synthetic tailings were conducted with raw seawater adjusted to the typical processing pH of 11, precipitated magnesium represented additional fine solids within the suspensions that competed with the tailings phases for the flocculant. This manifests in a reduced extent and efficiency of aggregation from the flocculation process; low settling rates in such tests indicate a reduced ability to recover water from gravity thickening. The use of seawater with a reduced magnesium concentration can reduce or even eliminate the formation of such precipitates, facilitating more effective aggregate forming interactions between flocculant and the tailings particles, reflected in greater aggregate size, higher settling rates, and more efficient fines capture.

The yield stress of concentrated suspensions formed after flocculation and sedimentation decreased significantly with an increasing proportion of treated seawater. This can be explained as a combination of several effects: (i) the system that has the least amount of magnesium requires a lower flocculant dosage to reach the established settling rate (20 m/h), which implies weaker particle networks, which then persist after sedimentation; (ii) the presence of precipitates represents an additional proportion of solids in the suspension that may be minor in mass, but their fine size heightens their impact on rheology. Reducing the magnesium content of seawater used in processing would therefore have advantages in terms of water return from thickening and subsequent underflow transport.

Treatment achieved high magnesium removal from seawater (Mg^2+^ 10 mg/L) and the settling rates attained for flocculated synthetic tailings in 100% treated seawater readily exceeded those that would be targeted for thickening. It, therefore, may not be necessary to arrange a complete removal to obtain acceptable industrial performance, and blending treated and raw seawater may be a practical option. However, this will depend on the specific tailings mineralogy.

Overall, the results confirm that the use of seawater, with a reduced magnesium content, has significant consequences on water and energy savings. The rheological properties are more favorable for pumping concentrated slurries when the magnesium content is reduced.

## Figures and Tables

**Table 1:** Solubility constant of precipitates.

Product	Solubility Product Constant (Kps)
$\mathrm{Mg}{\left(\mathrm{OH}\right)}_{2}$	$1.2\times 10^{-11}$
$\mathrm{Ca}{\left(\mathrm{OH}\right)}_{2}$	$4.1\times 10^{-6}$
${\mathrm{CaCO}}_{3}$	$6.7\times 10^{-7}$
${\mathrm{CaSO}}_{4}\cdot 2H_{2}O$	$3.14\times 10^{-5}$

**Table 2:** Experimental conditions of the eight systems prepared for rheological tests. Qz: quartz, Kao: kaolin, Rsw: raw seawater, Tsw: treated seawater.

Tailings 1: Qz/Kao 90/10		Tailings 2: Qz/Kao 75/25	
Type of Water (Rsw/Tsw)	Dosage (g/t)	Type of Water (Rsw/Tsw)	Dosage (g/t)
75/25	38	75/25	43
50/50	23	50/50	26
25/75	18	25/75	22
0/100	11	0/100	17

**Table 3:** Divalent cation concentration at pH 11.

Type of Water	Calcium (Ca^2+^)	Magnesium (Mg^2+^)
Raw seawater (Rsw)	335 mg/L	70 mg/L
Treated seawater (Tsw)	2210 mg/L	10 mg/L
Mixed seawater (Msw) (Tsw/Rsw = 50/50)	1215 mg/L	56 mg/L

## Data Availability

The data presented in this study are available on request from the corresponding author.
